# Ureteral dilatation detected in magnetic resonance imaging predicts vesicoureteral reflux in children with urinary tract infection

**DOI:** 10.1371/journal.pone.0209595

**Published:** 2018-12-21

**Authors:** Norihiro Murakami, Jun-ichi Kawada, Azumi Watanabe, Toshinao Arakawa, Takamasa Kano, Takako Suzuki, Ryo Tanaka, Daiei Kojima, Yoshihiko Kawano, Shin Hoshino, Hideki Muramatsu, Yoshiyuki Takahashi, Yoshiaki Sato, Masashi Koyama, Jun Natsume

**Affiliations:** 1 Department of Pediatrics, Nagoya University Graduate School of Medicine, Nagoya, Japan; 2 Department of Radiology, Okazaki City Hospital, Okazaki, Japan; 3 Department of Pediatrics, Toyota Memorial Hospital, Toyota, Japan; 4 Department of Pediatrics, Kasugai City Hospital, Kasugai, Japan; 5 Division of Neonatology, Center for Maternal–Neonatal Care, Nagoya University Hospital, Nagoya, Japan; 6 Brain and Mind Research Center, Nagoya University, Nagoya, Japan; 7 Department of Developmental Disability Medicine, Nagoya University Graduate School of Medicine, Nagoya, Japan; Yale University School of Medicine, UNITED STATES

## Abstract

**Objective:**

Urinary tract infection (UTI), one of the most common bacterial infections occurring during infancy and early childhood, is frequently associated with vesicoureteral reflux (VUR). Although several guidelines recommend performing ultrasonography as a screening test, its utility is not adequate and appropriate screening tests are strongly desirable. In this study, we evaluate the use of magnetic resonance imaging (MRI) as a screening test for VUR in children with UTI.

**Methods:**

We prospectively studied 108 patients with suspected UTI between April 2014 and March 2016. UTI was diagnosed on the basis of diffusion-weighted MRI (DW-MRI) and urine culture findings. We measured ureteral dilatation using MRI in 96 patients with UTI and assessed the relationship between ureteral dilatation in MRI and VUR in 46 patients who underwent voiding cystourethrography (VCUG).

**Results:**

Among 108 patients, 88 and 8 were diagnosed with upper and lower UTI, respectively. Among 46 patients who underwent VCUG, 23 had VUR (14 low grade and 9 high grade). Patients with ureteral dilatation detected on MRI had VUR more frequently than those without ureteral dilatation (any grades VUR, 71% vs. 32%; *P* = 0.02; high-grade VUR, 38% vs. 2%, *P* = 0.007). Overall, ureteral dilatation findings on MRI achieved sensitivity 65.2% and specificity 73.9% as a screening test for VUR. In addition, DW-MRI achieved sensitivity 100% and specificity 81.8% in the diagnosis of upper UTI.

**Conclusion:**

These findings suggested that MRI is a valuable tool for screening of VUR as well as diagnosis of upper UTI.

## Introduction

Urinary tract infection (UTI), one of the most common bacterial infections occurring in infancy and early childhood, is divided into two categories: “lower UTI” without renal inflammation and “upper UTI” with renal inflammation, including acute pyelonephritis (APN), acute focal bacterial nephritis (AFBN), and renal abscess.[1, 2] Because upper UTI frequently causes renal scarring and results in renal dysfunction, early diagnosis and early treatment are important.[3–5] However, diagnosis of upper UTI sometimes is challenging because of nonspecific clinical features, especially in infants.[6] Although urine culture is an important examination for diagnosis of UTI, upper and lower UTIs are difficult to distinguish on the basis of urine culture results. Furthermore, urine culture is considered negative in approximately 40% of patients with AFBN, which is a severe form of APN.[7] Therefore, negative urine culture results cannot rule out the possibility of upper UTI, and imaging tests, including ultrasonography (US), contrast-enhanced computed tomography (CECT), and ^99m^Technetium (^99m^Tc) dimercaptosuccinic acid (DMSA) renal scintigraphy, often are required to diagnose upper UTI.[8] In addition to these imaging procedures, some preceding studies showed the use of magnetic resonance imaging (MRI), especially diffusion-weighted magnetic resonance imaging (DW-MRI) in the diagnosis of APN.[9, 10]. Moreover, MRI is reportedly more accurate in the diagnosis of renal scarring compared with DMSA scan.[11]

Upper UTI frequently is associated with congenital anomalies of the kidney and urinary tract (CAKUT) such as vesicoureteral reflux (VUR) and pyeloureteral junction stenosis. VUR has been considered highly associated with renal scarring and renal dysfunction, and voiding cystourethrography (VCUG) is necessary for diagnosis of VUR.[12] However, the indication for VCUG should be determined carefully because of its invasiveness and radiation burden. Therefore, several guidelines recommend that VCUG should be performed only when CAKUT is strongly suspected.[2, 13] For example, the 2011 American Academy of Pediatrics clinical practice guidelines recommend to perform US in all patients with UTI at ages 2–24 months, whereas VCUG is recommended in patients with a second UTI episode or in those with abnormal US findings that suggest CAKUT, such as hydronephrosis and scarring.[2] However, preceding studies revealed that 30–50% of CAKUT cases could be overlooked by US alone,[14, 15] and appropriate screening tests to determine whether VCUG should be performed are strongly desirable.

To address issues, we evaluated the use of MRI for diagnosis of upper UTI as well as screening for CAKUT.

## Patients and methods

We prospectively enrolled 108 patients with suspected UTI (i.e., high fever and elevation of inflammatory markers without focus, high fever with pyuria, or any symptoms related to UTI, such as knocking pain of the costovertebral angle and dysuria) who were admitted to Nagoya University Hospital, Okazaki City Hospital, Konan Kosei Hospital, Japanese Red Cross Nagoya First Hospital, or Nakatsugawa City Hospital between April 2014 and March 2016. All patients underwent urine cultures obtained by transurethral bladder catheterization and blood cultures on admission. MRI was performed in all patients, whereas CECT, US, and VCUG were performed in 21, 21, and 46 patients, respectively. A positive finding was defined as pathogens isolated from urine samples with >5 × 10^4^ colony-forming units per milliliter or isolated from blood samples. MRI was performed using 1.5 Tesla MRI systems. All sequences were acquired during breath-hold except for DW sequences, which were acquired using respiratory trigger. We selected diffusion gradient b values as 0 and 1000 s/mm^2^. Administration of oral or rectal chloral hydrate was required in most infantile patients for mild sedation. All patients received standard maintenance fluid therapy, and daily fluid volume was determined by age and body weight (60–100 ml/kg/day). Initial MRI examinations were performed within 7 days after admission (median, 2 days after admission; range 0–7 days), and 42 of 108 patients underwent follow-up MRI examinations approximately 1 month after admission (median, 34 days; range, 15–70 days).

MRI images were evaluated by three expert radiologists. We defined renal lesions that were markedly hyperintense on DW-MRI and hypointense on apparent diffusion coefficient (ADC) map as pathological lesions.[16] The visibility of the renal lesions on DW-MRI and ADC map were quantified by assigning a score of 0–3 by each radiologist (0, no visible lesion; 1, probably no visible lesion; 2, probably with visible lesion; 3, with visible lesion) and summed the score of three radiologists (MRI score). We defined MRI score ≥5 as a positive finding. In addition, images with an MRI score of 4 were re-reviewed by the three radiologists, and we considered findings of images as positive if ultimate consensus was reached by all the three radiologists. We also evaluated the shape of the renal lesions and defined renal lesions with a long axis >20 mm as type A and others as type B lesions. Ureteral dilatation was evaluated by radiologists using T1-weighted, T2-weighted, and magnetic resonance urography (MRU) images and was classified into four categories (class 1, dilatation less than half of the ureter; class 2, dilatation more than half of the ureter; class 3, dilatation of the entire ureter without hydronephrosis; class 4, dilatation of the entire ureter with hydronephrosis). We defined classes 2–4 as positive ureteral dilatation findings.

We defined MRI positive with or without urine culture positive and MRI negative with urine culture positive as diagnostic criteria for upper and lower UTI, respectively. VUR was diagnosed and graded according to internationally accepted criteria.[17]

To compare the frequency of clinical features between the disease groups, we analyzed categorical variables using Fisher's exact test and continuous variables using the Mann–Whitney *U* test. All statistical analyses were performed using EZR (Saitama Medical Center, Jichi Medical University), which is a graphical user interface for R (The R Foundation for Statistical Computing, Vienna, Austria).[18]

Written informed consents were obtained from the parents of all the patients before participation in this study. This study was approved by the ethics committee of the Nagoya University Graduate School of Medicine and was conducted in accordance with the principles of the Declaration of Helsinki.

## Results

**Fig 1:**
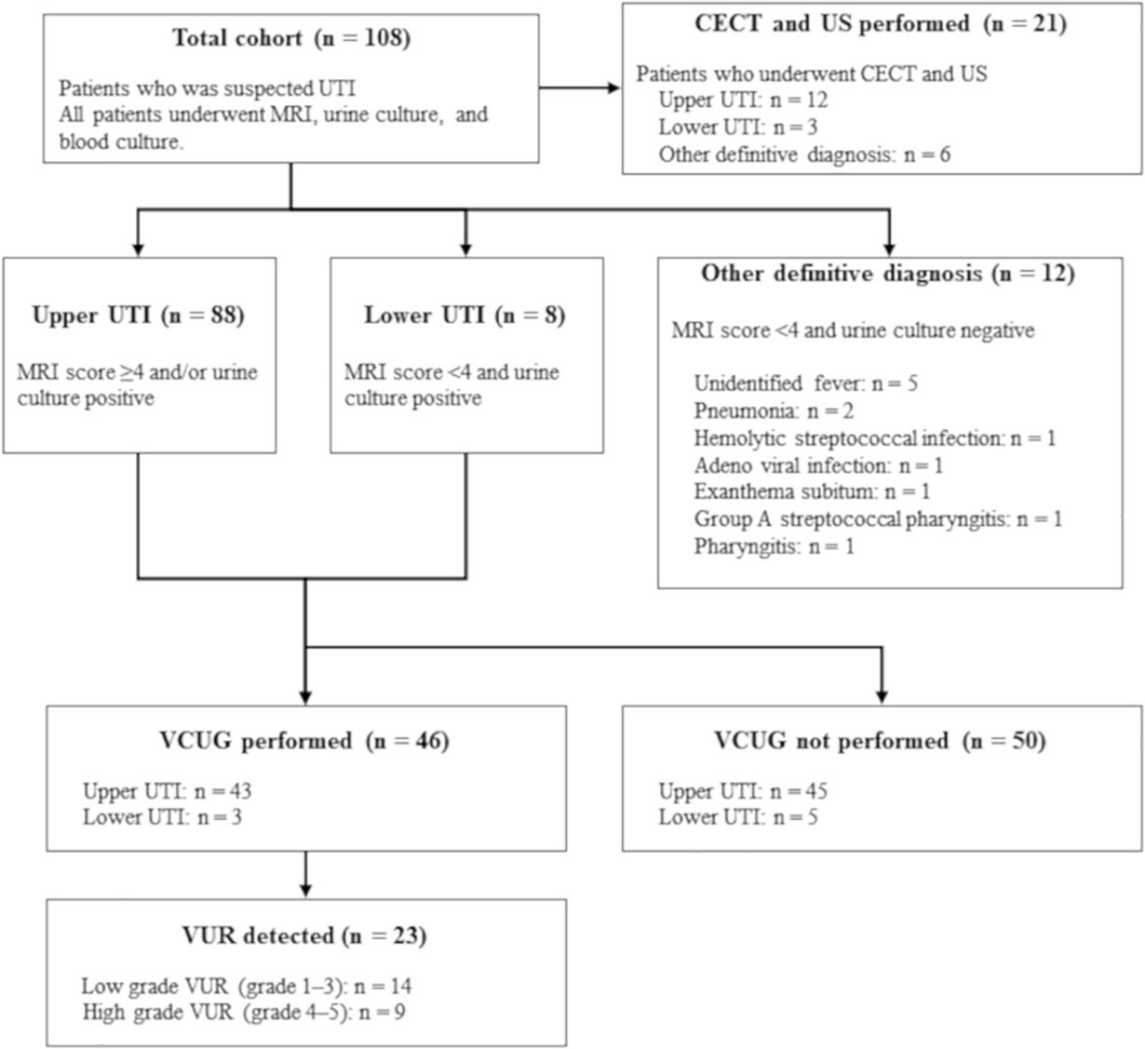
Flowchart showing formation of our study. UTI, urinary tract infection; MRI, magnetic resonance imaging; CECT, contrast-enhanced computed tomography; US, ultrasonography; VCUG, voiding cystourethrogram; VUR, vesicoureteral reflux.

Among 108 patients, 96 were diagnosed with UTI based on MRI score ≥4 and/or positive urine culture: 88 (MRI score ≥4) had upper and 8 (MRI score <4 and positive urine culture) had lower UTIs (Fig 1 and Table 1). Patients with upper UTI had higher C-reactive protein (CRP) levels (6.9 [0.8–28.7] vs. 2.7 [1.0–8.1] mg/dL; *P* = 0.0015) and longer duration of fever before admission (2 [1–8] vs. 1 [1–3] days; *P* = 0.06) compared with those with lower UTI (Table 2). Urine culture was negative in 43 (49%) patients with upper UTI, and the frequency of administration of oral antimicrobial agents before admission was not different between upper UTI patients with positive and negative urine cultures (5 of 45 vs. 8 of 43, *P* = 0.38).

**Table 1:** Patient characteristics.

	N = 108
Sex (male/female)	57/51
Age at admission, months, median (range)	7 (1–125)
Duration of fever before admission, days, median (range)	2 (1–9)
Diagnosis, n (%)	
Upper UTI	88 (81)
Lower UTI	8 (7)
Others	12 (12)
Pyuria at admission, n (%)	78 (72)
Bacteriuria at admission, n (%)	52 (48)
WBC at admission, ×10^9^/L, median (range)	17.8 (6.4–48.1)
Neutrophil at diagnosis, ×10^9^/L, median (range)	10.4 (3.5–34.6)
CRP at admission, ×10^9^/L, median (range)	6.3 (0.8–28.7)
Administration of antibacterial agents before admission, n (%)	18 (17)

UTI, urinary tract infection; WBC, white blood cell; CRP, C-reactive protein

**Table 2:** Differences between patients with upper UTI and lower UTI.

	Total	Diagnosis	
		Upper UTI	Lower UTI
	(n = 96)	(n = 88)	(n = 8)
Sex (male/female)	54/42	48/40	6/2
Age at admission, months, median (range)	7 (1–125)	7 (1–125)	5 (2–62)
Past history of UTI^a^	23	23	0
Duration of fever before admission, days, median (range) ^a^	2 (1–8)	2 (1–8)	1 (1–3)
Duration of fever after admission, days, median (range)	1 (1–4)	1 (1–4)	1 (1–3)
Urine culture positive, n (%)^a^	53 (55)	45 (51)	8 (100)
E.coli	45	39	6
E.faecalis	3	3	0
Others	5	3	2
MRI score, median (range)^a^	6 (1–9)	6 (4–9)	3 (1–3)
MRI ureteral dilatation findings, n			
class 1	62	55	7
class 2	19	18	1
class 3	6	6	0
class 4	9	9	0
VCUG performed, n (%)	46 (48)	43 (49)	3 (38)
VUR	23	23	0
grade 1–3, n	14	14	0
grade 4–5, n	9	9	0
WBC at admission, ×10^9^/L, median (range)	17850 (6400–48100)	17850 (6400–48100)	17950 (10600–26100)
CRP at admission, mg/dL, median (range)^a^	6.7 (0.8–28.7)	6.9 (0.8–28.7)	2.7 (1–8.1)

UTI, urinary tract infection; MRI, magnetic resonance imaging; VCUG, voiding cystourethrogram; VUR, vesicoureteral reflux; WBC, white blood cell; CRP, C-reactive protein

^a^*P* < 0.05 compared with lower UTI.

We evaluated the sensitivity and specificity of DW-MRI compared with CECT in 21 patients who underwent both imaging studies. Among the 21 patients, 10 had CECT-positive findings and an MRI score ≥4. Overall, DW-MRI achieved sensitivity 100%, specificity 81.8%, positive predictive value (PPV) 83.3%, negative predictive value (NPV) 100%, and diagnostic accuracy (DA) 90.5%. US also was performed in these patients, and only three with positive CECT findings showed positive US findings (hydronephrosis and renal enlargement in one, hydronephrosis in one, and low echoic lesions in renal parenchyma in one). US achieved sensitivity 30.0%, specificity 81.8%, PPV 60.0%, NPV 56.2%, and DA 57.1%.

**Fig 2:**
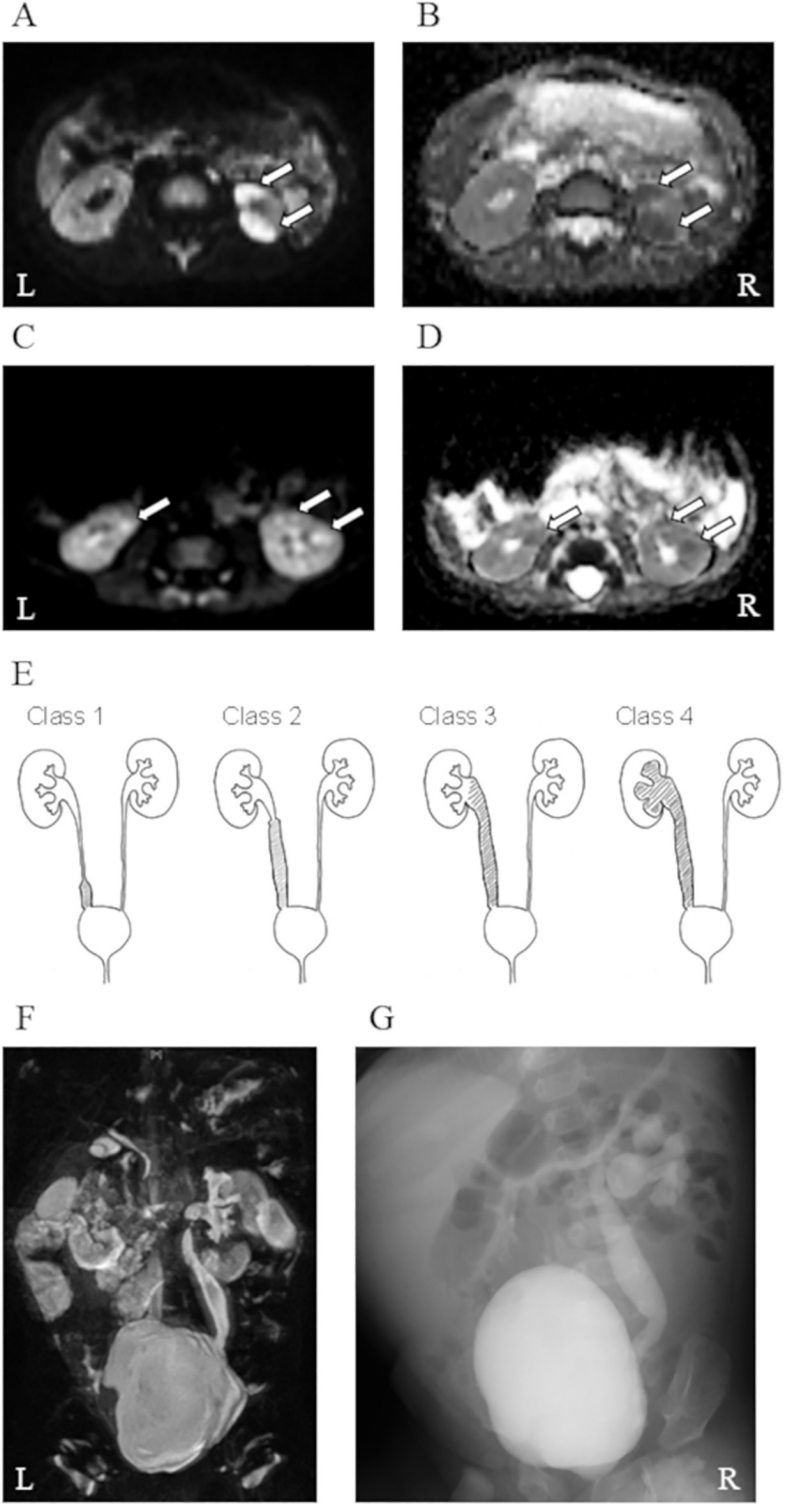
MRI findings. (A, B) Representative type A renal lesions detected by DW-MRI (A) and ADC map (B) in a 10-month-old boy. (C, D) Representative type B renal lesions detected by DW-MRI (C) and ADC map (D) in a 2-month-old boy. *Arrows* indicate renal lesions. (E) Schema of ureteral dilatation identified in MRI. Class 1, dilatation less than half of the ureter; class 2, dilatation more than half of the ureter; class 3, dilatation of the entire ureter without hydronephrosis; class 4, dilatation of the entire ureter with hydronephrosis. (F, G) A 5-month-old girl with upper UTI. MRU detected class 3 ureteral dilatation (F), and VCUG detected grade 4 VUR (G). DW-MRI, diffusion-weighted magnetic resonance imaging; ADC, apparent diffusion coefficient; MRI, magnetic resonance imaging; MRU, magnetic resonance urography; VCUG, voiding cystourethrogram; VUR, vesicoureteral reflux.

The shape of the lesions detected by DW-MRI was evaluated in 88 patients with upper UTI, and they were classified as type A (Fig 2A and 2B) in 63 and type B (Fig 2C and 2D) in 25 patients. Patients with type A lesions showed higher CRP levels (7.4 [1.3–28.7] vs. 5.3 [0.8–14.1] mg/dL, *P* = 0.01), higher MRI scores (8 [4–9] vs. 5 [4−7], *P* = 2.0 × 10^−5^), and longer duration of fever after admission (2 [1–4] vs. 1 [1−3] days, *P* = 0.01; Table 3) than patients with type B lesions. Among 88 patients with upper UTI, 42 underwent follow-up MRI approximately 1 month after admission (median 34 days; range, 15–70 days). Renal lesions identified on the initial MRI persisted on follow-up MRI in 37 of 42 (88%) patients.

**Table 3:** Differences between patients with type A lesions and type B lesions.

	Shape of the lesions	
	Type A	Type B
	(n = 63)	(n = 25)
Sex (male/female)	37/26	11/14
Age at admission, months, median (range)	7 (1–125)	6 (2–50)
Past history of UTI	15	8
Duration of fever before admission, days, median (range)	2 (1–8)	2 (1–6)
Duration of fever after admission, days, median (range)^a^	2 (1–4)	1 (1–3)
Urine culture positive, n (%)^a^	36 (57)	9 (36)
E.coli	30	9
E.faecalis	3	0
Others	3	0
MRI score, median (range)^a^	8 (4–9)	3 (1–3)
MRI ureter dilatation findings, n		
class 1	36	19
class 2	15	3
class 3	5	1
class 4	7	2
VCUG performed, n (%)	33 (52)	10 (40)
VUR	19	4
grade 1–3, n	11	3
grade 4–5, n	8	1
WBC at admission, ×10^9^/L, median (range)	18000 (6400–48100)	17800 (9400–34900)
CRP at admission, ×10^9^/L, median (range)^a^	7.4 (1.3–28.7)	5.3 (0.8–14.1)

UTI, urinary tract infection; MRI, magnetic resonance imaging; VCUG, voiding cystourethrogram; VUR, vesicoureteral reflux; WBC, white blood cell; CRP, C-reactive protein

^a^*P* < 0.05 compared with type B.

**Fig 3:**
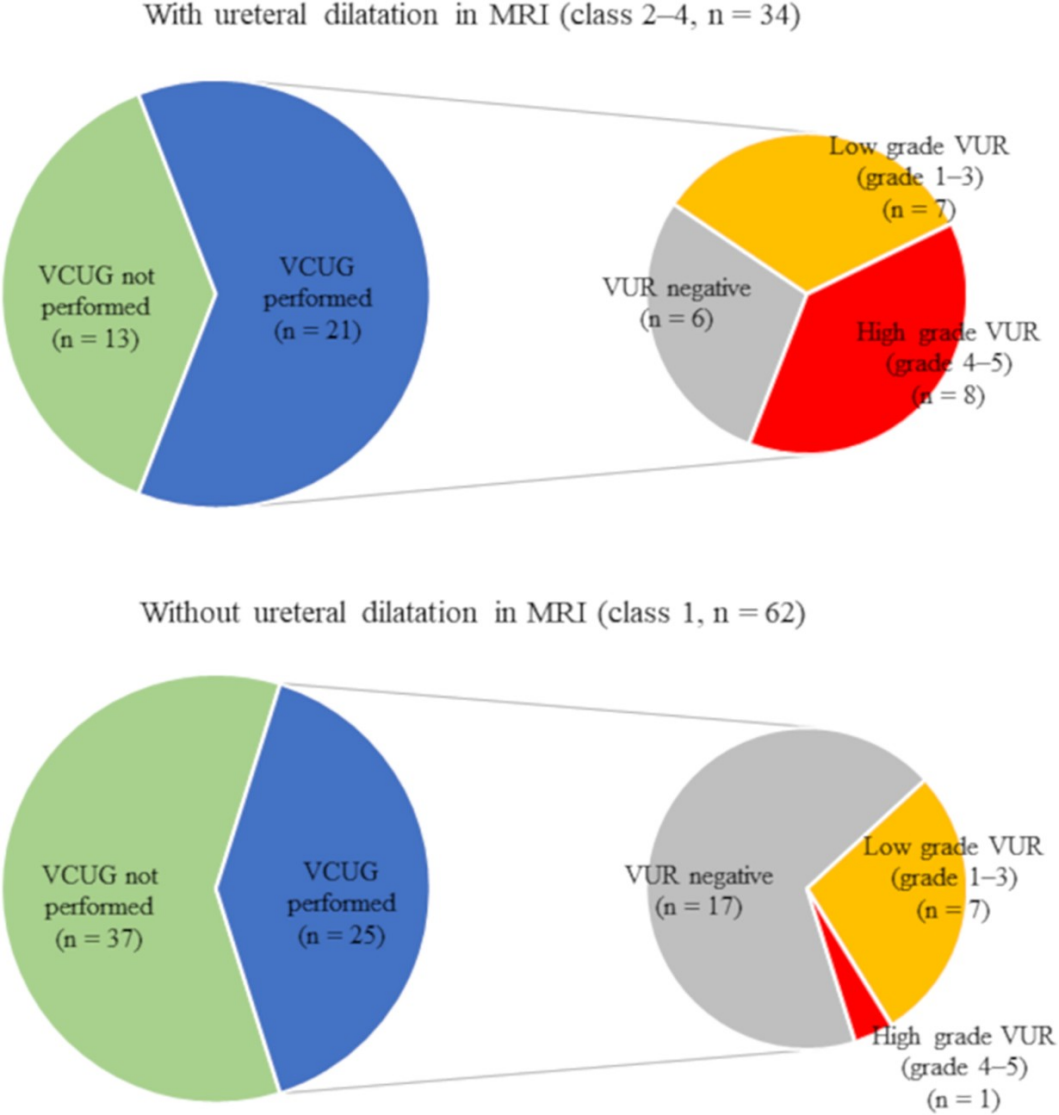
Relationships between ureteral dilatation findings in MRI and incidence of VUR. Each portion shows the number of patients with low-grade VUR, high-grade VUR, VUR negative among patients who underwent VCUG, and VCUG not performed in patients with or without ureteral dilatation in MRI. VCUG, voiding cystourethrogram; VUR, vesicoureteral reflux; MRI, magnetic resonance imaging.

Furthermore, we evaluated ureteral dilatation in 96 patients with upper and lower UTI and identified 62 with class 1, 19 with class 2, 6 with class 3, and 9 with class 4 dilatation (Fig 2E–2G). Class 2–4 ureteral dilatation was found in 33 (37%) patients with upper and 1 (12%) with lower UTIs (Table 2). VCUG was performed in 46 patients with UTI (43 upper and 3 lower UTIs), and VUR was identified in 23 (14 low grades 1–3 and 9 high grades 4–5 VUR). Interestingly, no patients with lower UTI harbored VUR, and there was no statistical difference in the frequency of VUR between urine culture positive and negative patients with upper UTI (10 of 25 patients with urine culture positive vs 13 of 21 patients with urine culture negative, *P* = 0.24). As shown in Fig 3, patients with class 2–4 ureteral dilatation detected by MRI had VUR more frequently than those without dilatation (15 of 21 [71%] vs. 8 of 25 [32%], *P* = 0.02). Furthermore, high-grade VUR (grade 4 or 5) was identified much more frequently in patients with ureteral dilatation (8 of 21 [35%] vs. 1 of 25 [2%], *P* = 0.007). Overall, class 2–4 ureteral dilatation findings detected on MRI achieved sensitivity 65.2%, specificity 73.9%, PPV 71.4%, NPV 68.0%, and DA 69.6% as a screening test for VUR and 88.9%, 64.9%, 38.1%, 96.0%, and 69.6%, respectively, as a screening test for high-grade VUR. On the other hand, US performed in 15 of 46 patients who underwent VCUG achieved 42.9%, 75.0%, 60.0%, 60.0%, and 60.0%, respectively, as a screening test for VUR and 60.0%, 80.0%, 60.0%, 80.0% and 73.3%, respectively, as a screening test for high-grade VUR.

## Discussion

We evaluated the use of MRI in children with UTI. To the best of our knowledge, this is the first study to reveal relationships between ureteral dilatation in MRI and VUR. We showed that ureteral dilatation findings detected on MRI at the acute phase of UTI were strongly related to the presence of VUR; therefore, MRI can be used as a screening test for VUR. In patients with UTI, US has been used commonly as a screening test for VUR, but it achieved a sensitivity of only approximately 40% in our study and 5–28% in a previous study.[15] Our data suggested that evaluation of ureteral dilatation using MRI is applicable to predict concurrent VUR, especially high-grade VUR whose surgical treatments occasionally were required. A recent study reported the association between ureteral dilatation measured by VCUG and the severity of VUR,[19] and this result might support our findings.

Diagnosis of upper UTI sometimes is difficult because urine culture is not always positive, especially in patients with AFBN.[7] Several previous reports showed that DW-MRI achieved excellent sensitivity and specificity (both over 90%) for diagnosis of upper UTI,[9, 10, 16] and we also showed equivalent performance compared with CECT, which is considered the best imaging procedure for diagnosis of AFBN. Conversely, US achieved a sensitivity of only approximately 30% in our study and a previous study.[8] These data suggested that DW-MRI, which can avoid radiation burden and administration of iodinated contrast, should be considered as an imaging test for children with suspected upper UTI. In addition, although urine culture was negative in about half patients with upper UTI in this study, the frequency of VUR was not different between urine culture positive and negative patients. This result may suggest patients who are MRI positive and urine culture negative should be considered as upper UTI.

We also showed that upper UTI patients with type A renal lesions on DW-MRI had higher CRP levels and MRI scores. They also had VUR more frequently than patients with type B renal lesions. This may suggest that type A lesion correlates with AFBN, which is a severe form of upper UTI.[7]

We showed that most renal lesions identified in the initial MRI persisted on follow-up MRI performed approximately 1 month after admission, despite improved clinical findings and inflammatory biomarkers. This result suggested that MRI might be used to evaluate the possibility of upper UTI in patients with undiagnosed fever retrospectively after healing. The appropriate follow-up timing of MRI in patients with upper UTI remains to be fully elucidated. In adult patients with APN, Faletti et al reported that 43 and 74% of APN foci detected by DW-MRI were radiological resolved at 1- and 3-month follow-up examinations, respectively.[20] Conversely, a preceding study reported that MRI could detect renal scar more than 6 months after UTI.[11] This result suggests that acute inflammatory renal lesions detected by DW-MRI persist for a long time as renal scars. However, further investigations are warranted to corroborate this finding.

In our cohort, all the patients with a previous history of UTI were diagnosed with upper UTI, and prior UTI might influence MRI score in such patients. However, there are no statistical differences in MRI score, WBC count, CRP, and duration of fever before admission between patients with upper UTI with or without a history of UTI. We believe that the effect of prior UTI on MRI findings is limited.

Finally, there are several limitations about our study. First, we could not enroll all patients with high fever and elevated inflammatory markers without focus during the study period. In our cohort, more than 90% of the UTIs were upper UTIs, but we could not deny that selection bias affected this result. In other words, this cohort preselected those children with a more severe presentation as children with milder upper UTI or lower UTI would potentially not have been admitted to hospital. Second, we performed CECT and US in a small number of patients. Therefore, comparison of use of MRI and CECT or US in patients with UTI was not sufficient.

In summary, we identified ureteral dilatation detected on MRI as related to the presence of VUR and showed the use of DW-MRI as a diagnostic imaging procedure for upper UTI in a prospective study. Our findings should contribute to the development of optimal therapeutic approaches in pediatric UTI.

## Supporting information

S1 File Demographics and clinical findings of individual patients. (XLSX)
